# High Concentrations of TNF-α Induce Cell Death during Interactions between Human Umbilical Cord Mesenchymal Stem Cells and Peripheral Blood Mononuclear Cells

**DOI:** 10.1371/journal.pone.0128647

**Published:** 2015-05-29

**Authors:** Xue Li, Wenjing Du, Feng Xia Ma, Xiaoming Feng, Francis Bayard, Zhong Chao Han

**Affiliations:** 1 The State Key Laboratory of Experimental Hematology, Institute of Hematology and Hospital of Blood Diseases, Chinese Academy of Medical Sciences and Peking Union of Medical College, Tianjin, China; 2 Institut de Médecine Moléculaire de Rangueil, Institut National de la Santé et de la Recherche Médicale, Toulouse, France; 3 National Engineering Research Center of Cell Products, AmCellGene Co. Ltd., TEDA, Tianjin, China; University of Cincinnati, College of Medicine, UNITED STATES

## Abstract

Human umbilical cord mesenchymal stromal cells (hUC-MSCs) are currently being used as novel therapeutic agents in numerous clinical trials. Previous works have shown that hUC-MSCs possess profound immunomodulatory capacities through IL-1 stimulation produced by peripheral blood mononuclear cells (PBMCs), their main cellular partner in most pathophysiological and therapeutic situations. The present study was designed to explore the role of TNF-α in these interactions. In these experiments, we demonstrated that TNF-α originated from PBMCs under the influence of IL-1. We also showed that TNF-α acted differently depending upon the concentrations reached. At low concentrations it clearly contributed to IL-6 and monocyte chemotactic protein 1 (MCP-1) production. At high concentrations, used alone or in association with the TNF-related apoptosis-inducing ligand, TNF-α also stimulated hUC-MSC IL-6 but, more intensely, MCP-1 production. This stimulation was associated but independent of apoptosis induction in a process involving Inhibitor of Apoptosis Proteins. Interferon gamma (IFN-γ), tested to stimulate PBMC and tissue activation, amplified IL-6 and MCP-1 production and cell death by, apparently, a different process involving necrosis. Our findings bring new insights into the complex interactions between hUC-MSCs and PBMCs, involving cytokines, chemokines and cell death, and are of fundamental importance for tissue homeostasis.

## Introduction

Mesenchymal stem cells, better denoted as multipotent mesenchymal stromal cells (MSCs) [1], are the focus of intense efforts at elucidating their nature and unique properties as well as developing cell-based therapy for a diverse range of diseases ([2–4] and references therein). MSCs have been isolated from many different tissues, including bone marrow, adipose tissue, umbilical cord, amniotic fluid, and placenta. Apparently, all share many common characteristics, amongst which are their profound anti-immunosurveillance properties and stimulation of tissue regeneration through secretion of therapeutic factors [5]. Several factors or cytokines have been implicated in the immunoregulation of MSCs, such as IDO, IL-10, TGFβ, TSG6[6].

Human umbilical-cord-derived mesenchymal stromal cells (hUC-MSCs), which can be isolated and expanded easily in large quantities *in vitro*, are being explored as promising candidates for potential clinical applications [6, 7]. We previously demonstrated that, by their PGE2 production, hUC-MSCs played an important role in inhibiting proliferation and interferon gamma (IFN-γ) secretion by lymphocytes in response to mitogenic or allogeneic stimuli. CD14+ monocyte-secreted Interleukin-1β (IL-1β) was involved in PGE2 and IL-6 secretion, although this cytokine appeared not to be involved in the T cell suppression but was active in generating immunosuppressive macrophages [8–12].

The therapeutic application of MSC may require cell delivery or migration to sites of inflammation and the potential immune-regulation of MSC under these condition is not clear. In the present studies, we focused on TNF-α to clearly define its role in the communication between hUC-MSCs and the peripheral blood mononuclear cells (PBMCs) in wound healing. Interleukin-6 (IL-6) and chemokine ligand-2/monocyte chemiattractant protein-1 (MCP-1) were used as relevant markers of hUC-MSCs stimulation. We demonstrated that TNF-α production originates in PBMCs under the influence of IL-1. We also showed that, in high concentrations, TNF-α stimulated hUC-MSC production of IL-6 but, to a greater extent, MCP-1 that could be associated with induction of cell death in a process involving Inhibitor of Apoptosis Proteins (IAPs) but independent of the cell death itself. This effect was increased by the presence of TNF-related apoptosis-inducing ligand (TRAIL) and INF-γ.

## Materials and Methods

### Origin, isolation and *ex vivo* expansion of hUC-MSCs

This study was approved by the Institutional Review Board of Chinese Academy of Medical Sciences and Peking Union Medical College. Umbilical cords and peripheral blood were obtained from donors with written informed consent. hUC-MSCs were isolated from umbilical cords obtained from local maternity hospitals. Isolation, *ex vivo* expansion and characterization of hUC-MSCs were essentially as described previously [13]. Passages 4 to Passages 10 hUC-MSCs were used in this study.

Isolation of human PBMCs and preparation of conditioned supernatant (SN) have been previously described [8, 9].

### Media and reagents

PBMCs and hUC-MSCs were grown in DMEM/F-12 (Invitrogen) supplemented with 10% FCS (Hyclone), 2 mM glutamine, 100 U/ml penicillin and streptomycin, 1 mM sodium pyruvate and 10ng/ml hEGF (Peptrotech). hUC-MSCs were harvested using trypsin/EDTA. TNF-α, IL-6, IFN-γ, FasL, IL-1ra and TRAIL were purchased from PeproTech. IL-1β was from R&D. LY2940002, JNK inhibitor II (CAS 129-56-6), BAY 11–7082 and SC-514 were purchased from Calbiochem. U0126 and SB203580 were purchased from Sigma-Aldrich. GDC-0152 was purchased from Selleck.

### Cytokine stimulation

For hUC-MSCs, hUC-MSCs(2×10^4^/well) were cultured in 96-well plates for 18 hours. Then, exogenous IL-1β (10ng/ml) or TNF-α (5, 10, 20 ng/ml) or TRIAL(500ng/ml) or IFN-γ(50ng/ml) were added to the hUC-MSCs. For experiment using inhibitors, hUC-MSCs were pre-treated with related inhibitors for 2 hours, then treated with stimulators, TNF-α, IFN-γ or TRAIL. The SN was then collected at specified time and cytokine levels detected.

### Determination of cytokine concentrations by enzyme-linked immunosorbent assays (ELISA)

Cell-free supernatants were collected and kept frozen at -80°C until assayed for cytokine concentrations by ELISA. ELISA assay kits for TNF-α IL-1β, IL-6 and MCP-1, were used following the instructions of the supplier (NeoBioscience Technology Company, P.R. China).

### Luminescent Cell Viability Assay

hUC-MSCs were seeded into 96-well plates at a concentration of 2×10^4^ cells /well and treated with different concentrations of cytokines (20ng/ml TNF-α, 500ng/ml TRIAL or 50ng/ml IFNγ) with or without 20 μM z-VAD-fmk (Promega) or 50 μM necrostatin-1 (Sigma) for 24 or 48 hours. Cell viability was measured using the CellTiter Glo Luminescent Cell Viability Assay (Promega) according to the directions of the manufacturer. After measurement by Gen5 (Biotect), results were analyzed by the related software.

### Western Blotting

Protein from nucleus and cytoplasma were extracted separately by Nuclear and Cytoplasmic Protein Extraction Kit(Beyotime, Shanghai, China). Equal amounts of protein extracts were separated by SDS-PAGE gel and transferred electrophoretically to PVDF membranes (Millipore, USA). The membranes were blocked in TBST containing 5% skim milk at room temperature for 2 hours. After washing with TBST, the blocked membranes were probed with anti-NF-kB p65(Santa Cruz 1:200), anti-β-actin (Santa Cruz,1:1000) overnight at 4°C, and subsequently washed with TBS containing 0.1% Tween-20. After washes, the membranes were incubated with goat anti-rabbit horseradish peroxidase-conjugated secondary antibodies (Santa Cruz 1:3000) for 1 hour at room temperature. The secondary antibodies were detected with the Western chemiluminescent ECL reagent (Thermo Scientific Pierce). The Integrated Density of protein was quantified by Image J software.

### Statistical analysis

Statistical analysis was carried out using the GraphPad Prism 6 software (Version 6.0b, October 3, 2012). Data are presented as means±SEM, and p<0.05 was considered to be statistically significant.

## Results

### TNF-α was produced by PBMCs in an IL-1-dependent fashion

**Fig 1:**
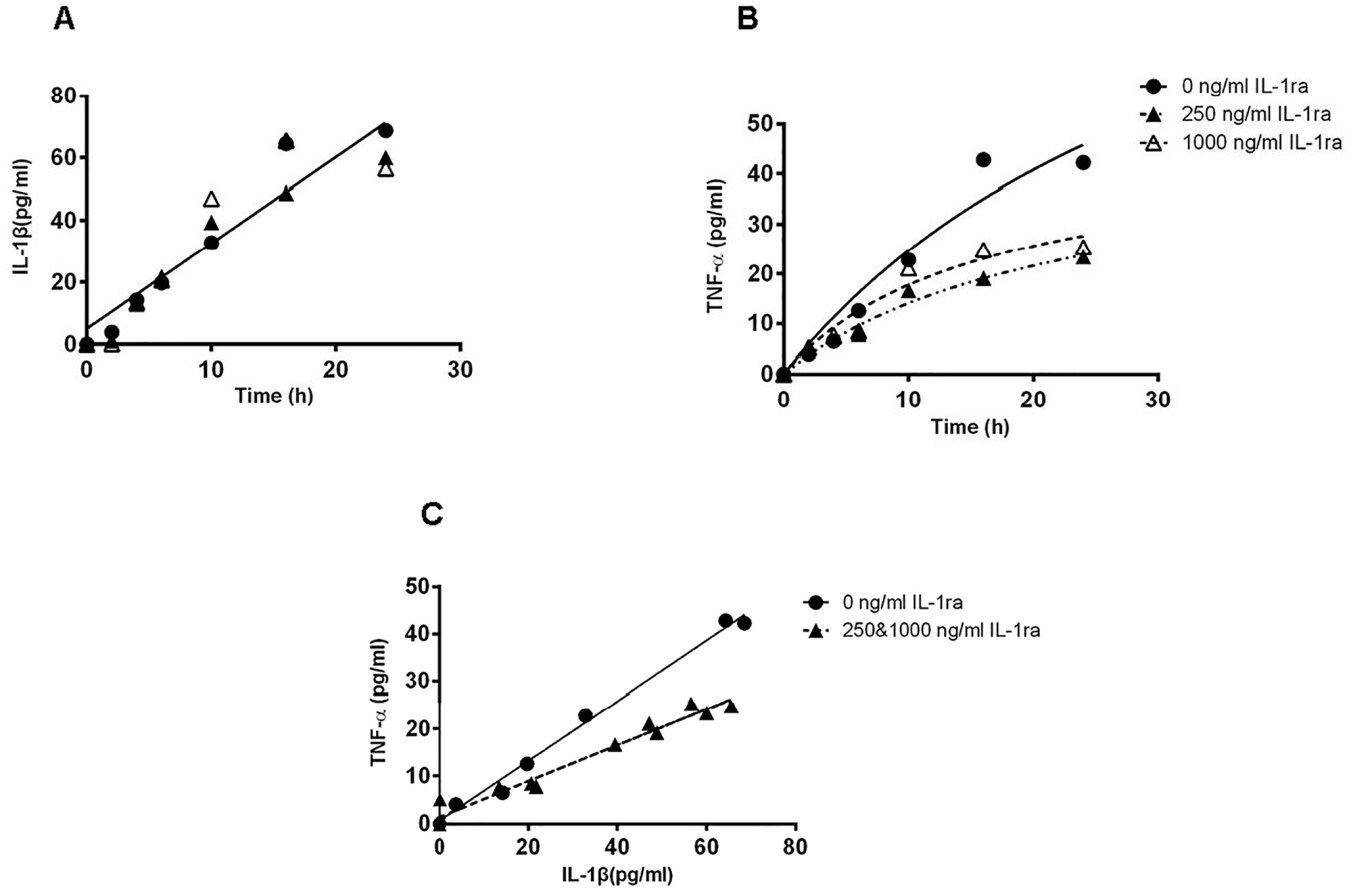
TNF-α was produced by PBMCs in an IL-1ß-dependent fashion. IL-1ß(A) and TNF-α(B) concentrations were measured in PBMC SN treated without or with IL-1ra (250 and 1000 ng/ml; 15 and 59 nM). IL-1ra was added at time 0 and SN collected at times 2, 4, 6,10, 16 and 24h. The relationship between TNF-α and IL-1ß concentrations were measured in the same samples obtained at the different times in absence or presence of IL-1ra. Data from 250 and 1000 ng/ml IL-1ra-treated samples were pooled (Fig 1C). Data are expressed as pg/ml from 2x10^4^ cells in 96 well plates. This experiment has been repeated twice.

TNF-α was not detected in hUC-MSC SN neither in basal nor IL-1 stimulated conditions but could be detected in PBMC SN, suggesting that TNF-α originated from PBMCs in parallel with IL-1ß production. As shown in Fig 1A, IL-1ß concentrations measured in PBMC SN as a function of time increased linearly up to 24h and were not affected by the presence of IL-1ra. Analyzing the equation of curve generated by the least squares fitting method, showed that a linear regression was observed for the 3 curve obtained in absence or presence of the 2 concentration of IL-1ra. Moreover comparison between these 3 curves by Extra sum-of-squares F test excluded the possibility that different curves were necessary to represent the different data sets(p = 0.66). One single common straight line (r^2^ = 0.83) adequately fitted all the data sets. This demonstrated that IL-1ra had no influence on IL-1ß production by PBMCs.

For TNF-α, using similar statistical method analysis, the time-production relationship in absence of IL-1ra was hyperbolic (r^2^>0.9). In presence of IL-1ra, the relationship was also hyperbolic(r^2^ = 0.86 and r^2^ = 0.89, in presence of 250 or 1000ng/ml IL-1ra). However, comparison of the 3 curves obtained in absence or presence of IL-1ra could not be represented by a same curve (p<0.001). The effect of 250 ng/ml IL-1ra (15 nM) was as effective as 1000 ng/ml (59 nM) and these two series of data could be represented by the same hyperbolic curve (r^2^ = 0.83). Concentrations measured at 24h, which reflect the endogenous production of PBMCs over the total period of the studies, were lowered by half when compared with IL-1ra-untreated PBMCs (unpaired t test, p<0.005). Finally, we analyzed the relationship between TNF-α and IL-1β concentrations measured in the same samples obtained at the different times of the studies in absence or presence of IL-1ra, pooling together 250 and 1000ng/ml IL-1ra-treated samples (Fig 1C). A highly significant correlation was observed (Pearson r = 0.99 and 0.98 in absence or presence of IL-1ra respectively). These whole series of data confirmed that TNF-α production was dependent upon IL-1ß production.

### TNF-α and IL-1ß induced different activities in IL-6 and MCP-1 production that were increased by IFN-γ

**Fig 2:**
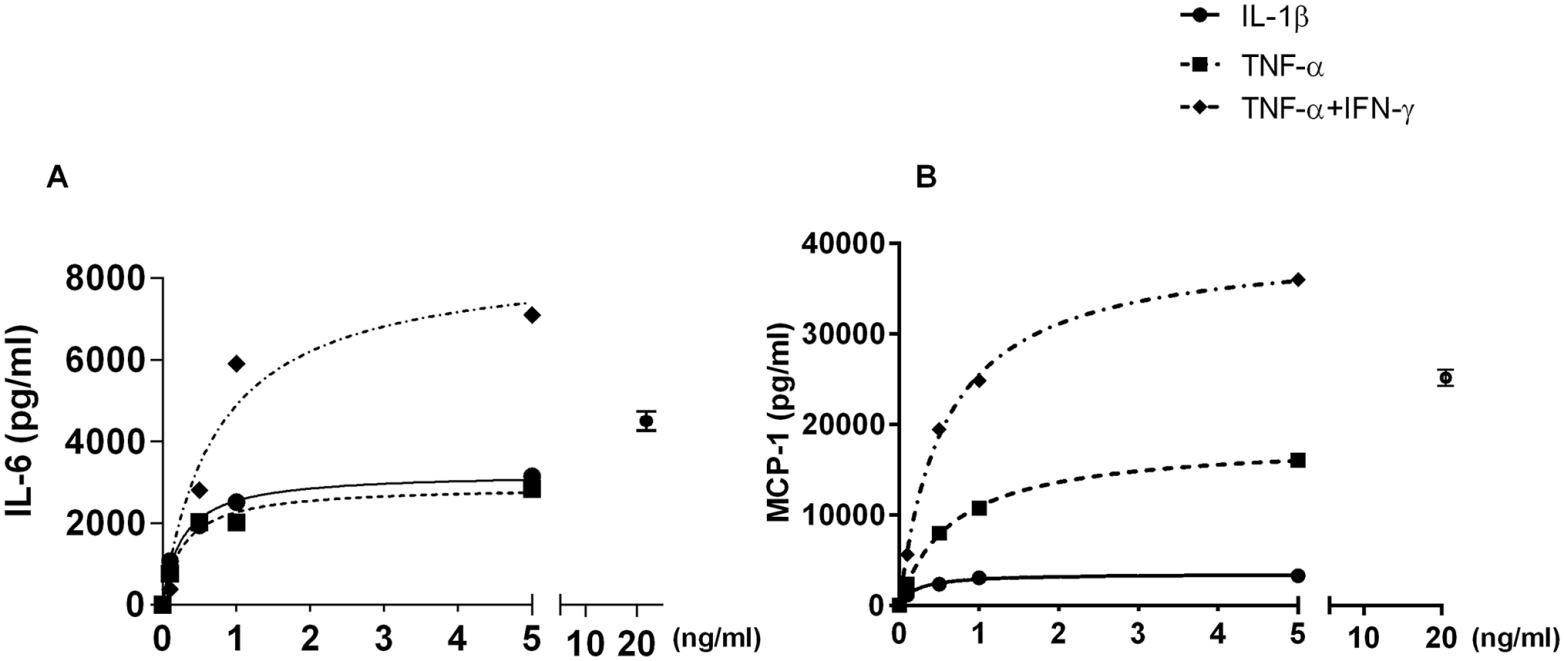
IL-6 and MCP-1 production as a function of IL-1ß and TNF-α concentrations in absence or presence of IFN-γ. IL-6 (A) or MCP-1 (B) concentration were measured after MSCs were treated with different concentration of TNF-α or IL-1β with or without IFNγ (50ng/ml). To precisely analyze the stimulating activities, basal Il-6 and MCP-1 expression (without treatment) have been subtracted from the concentrations measured under TNF-α or IL-1β with or without IFNγ stimulation. These basal concentrations were in the range of 10 to 20% of the maximal stimulating effect for IL-6 and in the range of 33% for MCP-1. The data are representative of 2 separate experiments. One over 3 measurements have been plotted for the clarity of the figures. The concentrations measured under 20 ng/ml concentration are represented as a single point (± SEM) as they did not fit with the one binding hyperbola which fitted all other concentrations obtained at different TNF-α concentrations.

Recombinant TNF-α (0 to 5 ng/ml; 0–0.28 nM) induced IL-6 production by hUC-MSCs (Fig 2) that followed a hyperbolic dose-response curve (r^2^ = 0.98) with a half-maximal stimulation at 0.3 ng/ml TNF-α (0.016±0.005 nM, mean±SEM). This dose-response curve was similar to the curve obtained for IL-1ß stimulation. When combined with IFN-γ (50 ng/ml (3 nM), a concentration measured when PBMCs were stimulated by phytohemagglutinin [8–10]), TNF-α induced increased IL-6 production along a hyperbolic relationship (r^2^ = 0.95). Interestingly, the half-maximal stimulation was increased and reached at 0.7 ng/ml TNF-α (0.044±0.019 nM, mean±SEM). This contrasted with what was observed when IFN-γ was combined with IL-1ß where a half-maximal stimulation decreased from 0.13 ng/ml to 0.05 ng/ml IL-1ß, suggesting an amplification of the IL-1ß signaling pathway.

Observations regarding MCP-1 production revealed a peculiar stimulation scheme. In contrast to the IL-6 production where IL-ß and TNF-α activities were similar (considering cytokine concentrations or molarities), TNF-α was more effective than IL-1ß on MCP-1 production. Its effect was also increased when IFN-γ was added.

We previously reported that JNK and NF-kB signaling pathways were activated under Il-1β stimulation[8, 9]. We, then, evaluated the signaling pathway involved with TNF-α stimulation. The effect of cell permeable signaling inhibitors of PI3K (LY294002), c-JNK-1, 2 and 3 (CAS 129-56-6), MEK1 and MEK2 (U0126) and of the p38 MAP kinase (SB203580), all used at submaximal concentrations of 10 μM to amplify possible differences, were then studied. It was observed that the signaling pathways involved in IL-6 production, essentially p38 MAP kinase-dependent, was different than under IL-1ß stimulation, essentially JNK-dependant. Moreover, for MCP-1 production, the PI3K and c-JNK signaling pathways appeared dominant (S1 Fig). NF-kB was, nevertheless, constantly effective on both IL-6 and MCP-1 production as shown by the effect of the IKK inhibitors Bay 11–7082 and SC-514 (S2 Fig).

Moreover, when higher TNF-α concentrations were used to stimulate IL-6 and MCP-1 production, a new phenomenon was observed. Although the maximal concentration expected for IL-6 from the dose-response curve was 2900 ng/ml (Fig 2A), the measurements showed a concentration of 4321±416 pg/ml at 20 ng/ml TNF-α, significantly higher than the concentration measured at 5 ng/ml (2843±59 pg/ml IL-6; p<0.05 by unpaired t test). At 10 ng/ml TNFa (2937±137 pg/ml), the IL-6 concentration was not significantly different from the 5 ng/ml point but lower than the 20 ng/ml point (p<0.05). Similarly, for MCP-1, for an expected maximal concentration of 18000 pg/ml (Fig 2B), the measurements showed a concentration of 22855±416 pg/ml at 20 ng/ml, significantly higher than the concentration measured at 5 ng/ml TNF-α (16040±1841 pg/ml; p<0.05) but not at 10 ng/ml (17412±937 pg/ml; p = 0.0579). Similar observations were also repeatedly observed when TNF-α was associated with IFN-γ stimulation. Besides the involvement of unusual signaling pathways for TNF-α activity in presence or absence of IFN-γ, these series of data suggested a new mechanistic pathway that also ended in NF-kB.

### TNF-α induced hUC-MSC death that was amplified by TRAIL and IFN-γ

TNF-α exerts its biological effects interacting with two different receptors: TNFR1 and TNFR2 and two different signaling pathways, one triggering cell death processes and the other, the NF-kB pathway, controlling expression of inflammatory and anti-apoptotic proteins.

**Fig 3:**
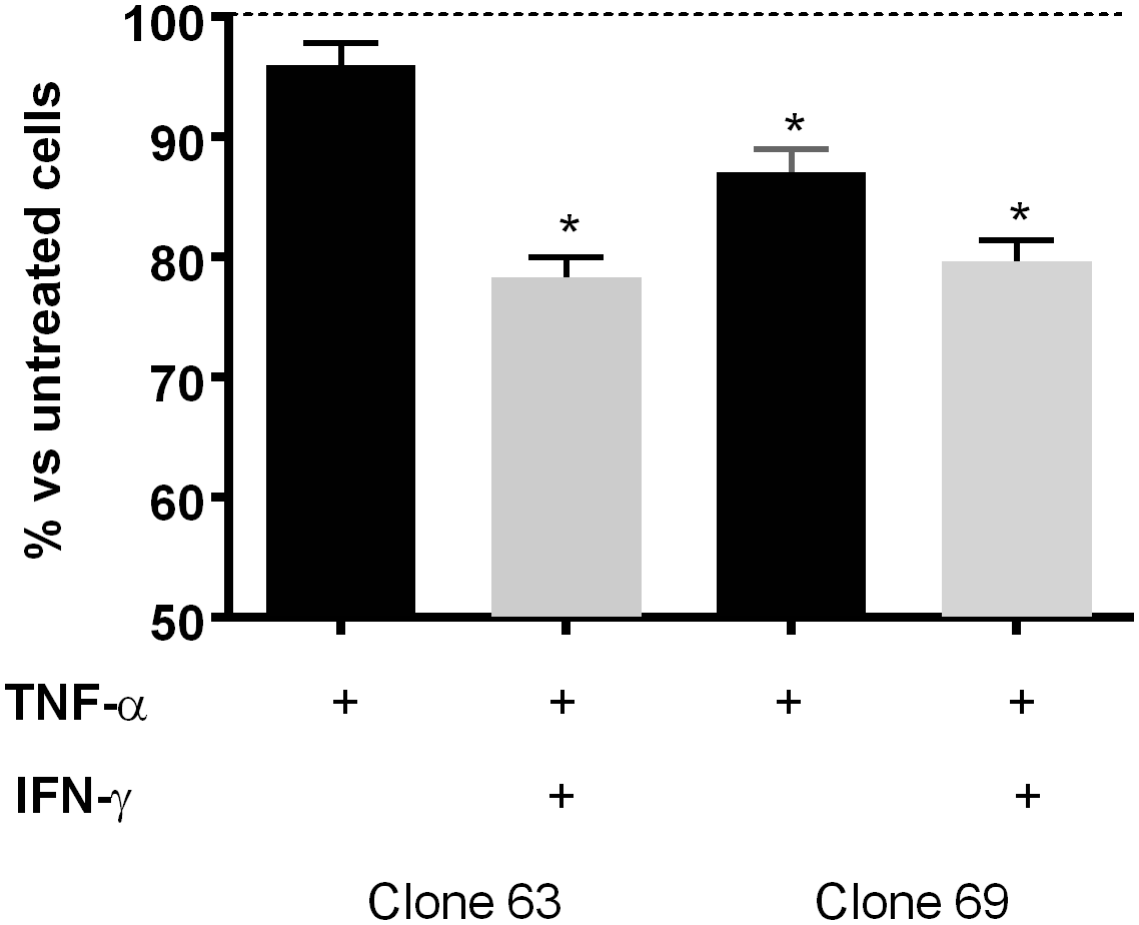
TNF-α-induced hUC-MSC death amplified by IFN-γ. hUC-MSCs of two different clones (Clones 63 and 69, 2x10^4^ in 96-well plates) were stimulated with TNF-α (20 ng/ml, 1.2 nM) or TNF-α associated with IFN-γ (50 ng/ml, 3 nM) for 48h. Then, cell death was scored by CellTiter-Glo Luminescent Cell Viability Assay (mean±SEM of triplicate ATP measurements expressed as % of control untreated cells; * p<0.001 when compared with untreated cells). Representative of 3 different experiments.

The CellTiter-Glo Luminescent Cell Viability Assay was used to measure ATP as an indicator of viable cells following TNF-α stimulation. Using different hUC-MSCs clones, early passages (2 to 4) appeared more sensitive than later passages (>7) to cell death induction. Time course analysis from 24 to 72h showed that measurements were more stable after 48h than after 24, suggesting a degree of asynchrony within the hUC-MSC population. In these conditions, 20 ng/ml (1.1 nM) TNF-α appeared to induce a maximal activity on sensitive clones although, as shown in Fig 3, some clones were more sensitive than others. However, a significant and constant cell death inducing activity was observed when IFN-γ was associated with TNF-α. The effect of the association appeared not as marked when TNF-α was already active by itself (clone 69 as an example).

**Fig 4:**
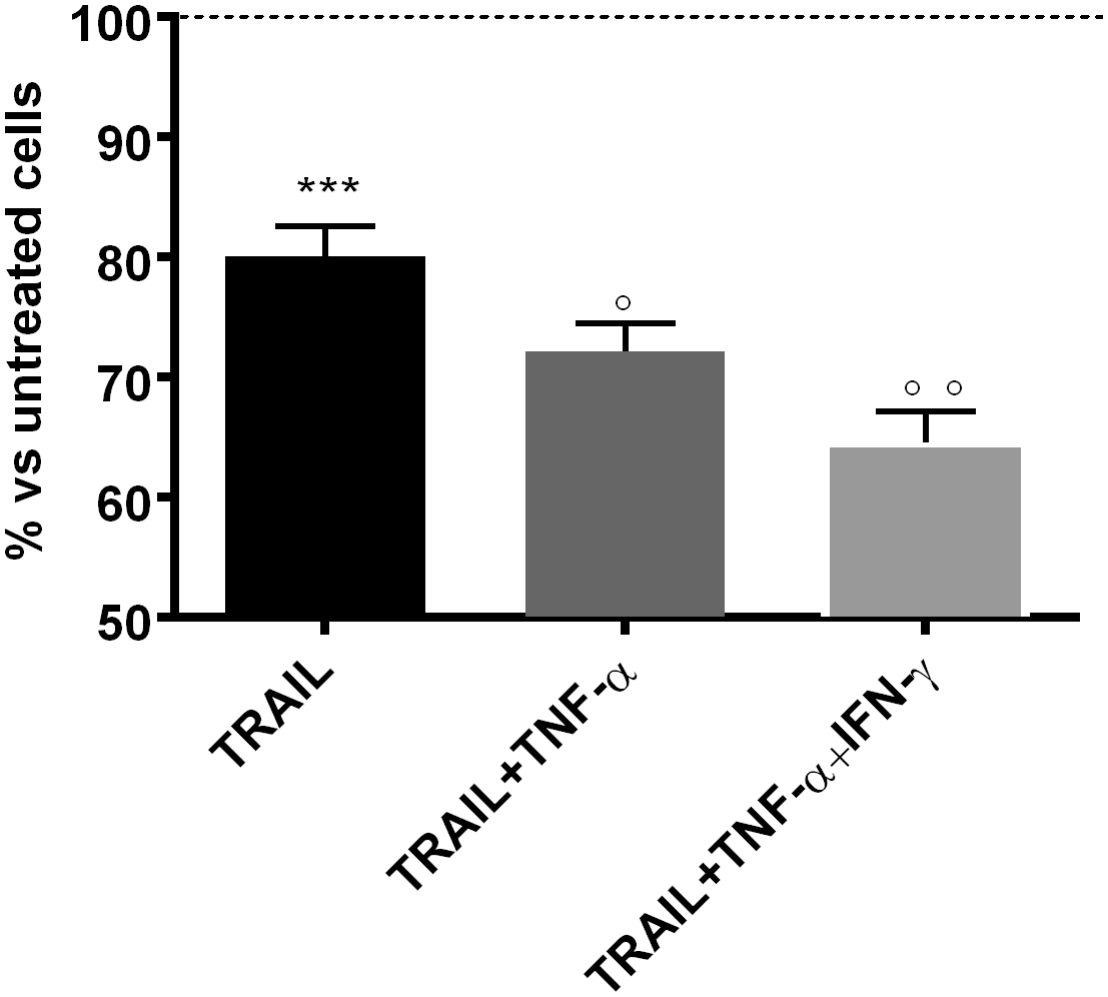
TNF-α-induced hUC-MSC death amplified by TRAIL. hUC-MSCs of two different clones (Clones 63 and 69, 2x10^4^ in 96-well plates) were stimulated with TRAIL (500 ng/ml, 28 nM) alone or in association with TNF-α (20 ng/ml, 1.2 nM) and IFN-γ (50 ng/ml, 3 nM) for 48h. Then, cell death was scored by CellTiter-Glo Luminescent Cell Viability Assay. Data from the two clones were pooled (mean±SEM of six ATP measurements; *** p<0.0001 when compared with untreated cells; °p<0.05, °°p<0.01 when compared with TRAIL alone-treated cells). Representative of 3 different experiments.

Association of TNF-α with one of the death receptor ligands FasL or TRAIL using recombinant soluble recombinant forms was studied. FasL (500ng/ml) had no effect by itself and was not reproducibly active in association with TNF-α. In contrast, TRAIL used at the same concentration (500ng/ml, 28nM) was active by itself (Fig 4) and its effect was increased in presence of TNF-α (p<0.05 by unpaired t test) and a mixture TNF-α and IFN-γ (p<0.01).

**Fig 5:**
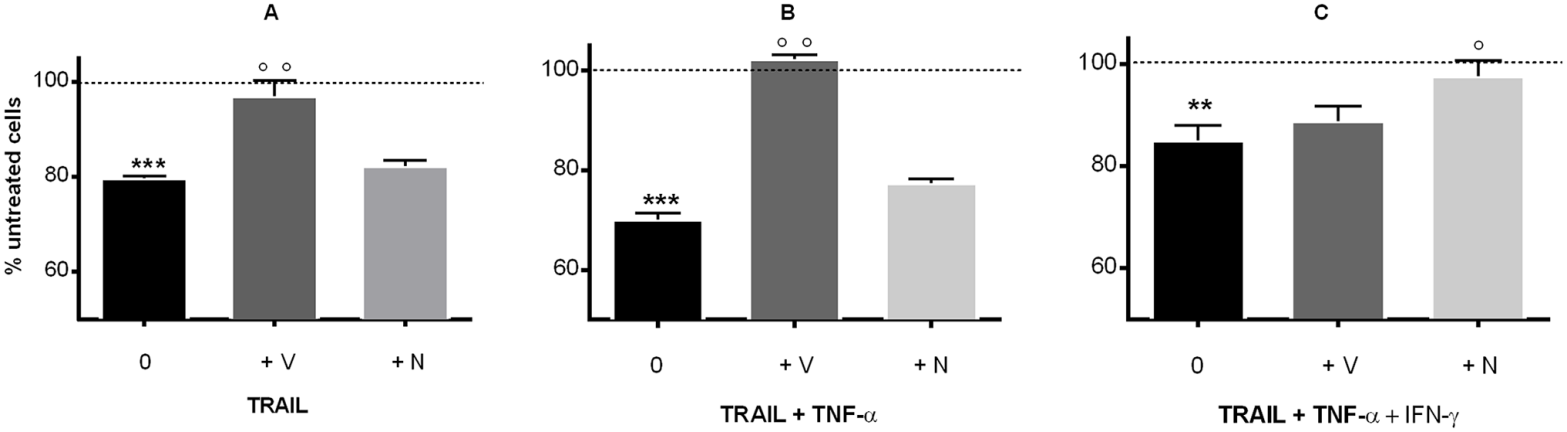
Mechanisms involved in cell death. hUC-MSCs of clone 63 (2x10^4^ in 96-well plates) were pretreated for 2h with zVAD-fmk (V, 20 μM) or necrostatin-1 (N, 50 μM) then stimulated with TRAIL (500 ng/ml, 28 nM) alone or in association with TNF-α (20 ng/ml, 1.2 nM) and IFN-γ (50 ng/ml, 3 nM) for 24h. Then, cell death was scored by CellTiter-Glo Luminescent Cell Viability Assay. Data are presented as mean±SEM of three ATP measurements; *** p<0.0001, **p<0.01 when compared with untreated cells; °p<0.05, °°p<0.01 when compared with TRAIL-treated, TRAIL-associated with TNF-α or TNF-α associated with IFN-γ-treated cells). Representative of 2 different experiments using alternatively hUC-MSCs clone 63 and 69 with the same results.

In order to characterize the mechanisms involved in cell death, we explored the inhibition of caspase activity using the broad-spectrum caspase inhibitor zVAD-fmk (20μM) and the specific inhibitor of RIPK1 kinase activity Necrostatin 1 (50μM). In these experiments, incubation was pursued for 24h instead of 48h to prevent a toxic effect of the inhibitors. As shown in Fig 5, cell death resulted from an apoptotic phenomenon with TRAIL and TNF-α treatments but from a necrotic phenomenon when IFN-γ was present.

### MCP-1 and, to a lesser extent, IL-6 production were indirectly linked to cell death and involved inhibition of apoptosis proteins

**Fig 6:**
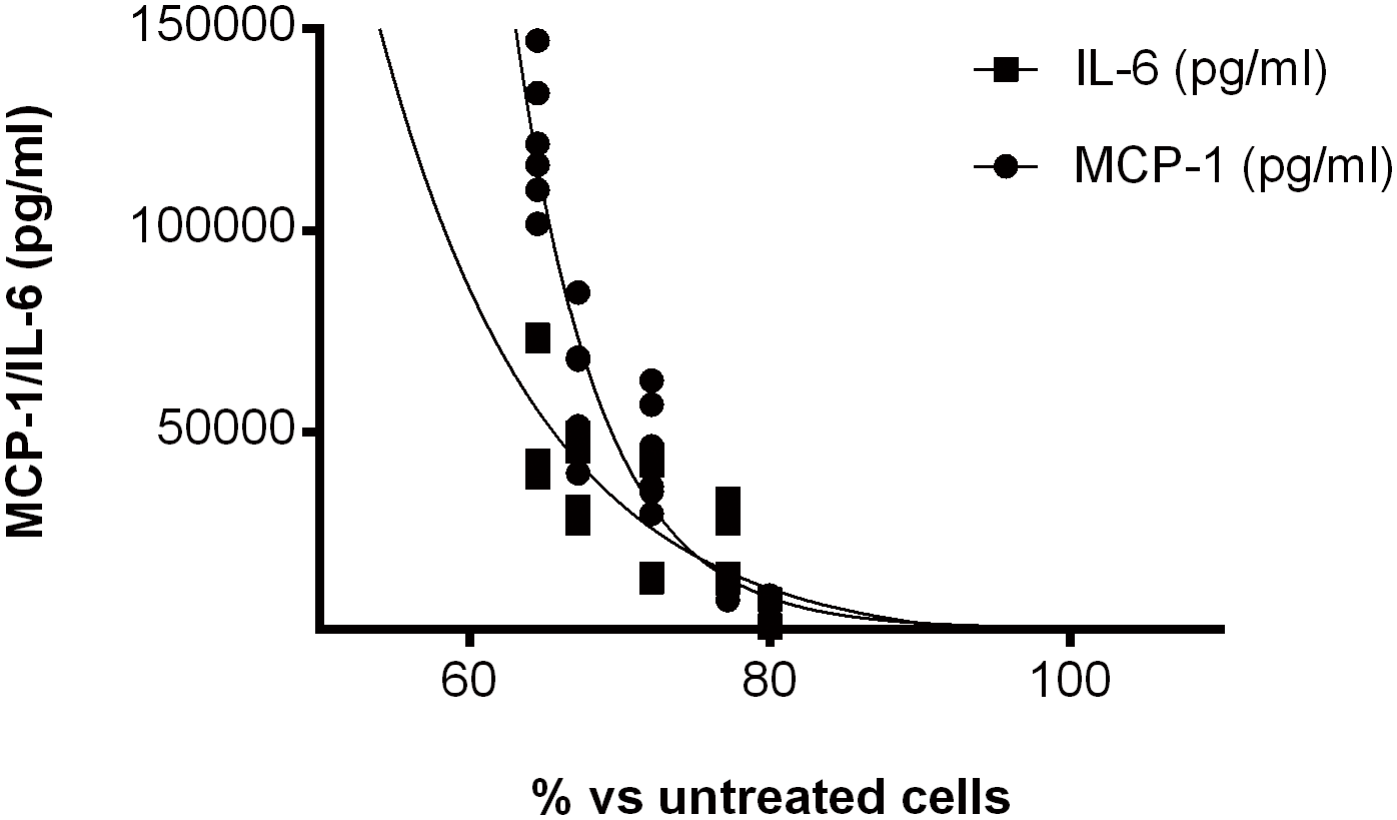
Relationship between cell death and cytokine concentrations. hUC-MSCs of different reactive clones (2x10^4^ in 96-well plates) were stimulated with TNF-α (20 ng/ml, 1.1 nM), TRAIL (500 ng/ml, 28 nM) alone or in association with TNF-α or TNF-α associated with IFN-γ (50 ng/ml, 3 nM) for 48h. Then, cell death was scored by CellTiter-Glo Luminescent Cell Viability Assay and expressed as % of control untreated cells. A statistically significant one-phase decay was observed for both IL-6 and MCP-1 with r^2^ = 0.74 and 0.92 respectively.

**Fig 7:**
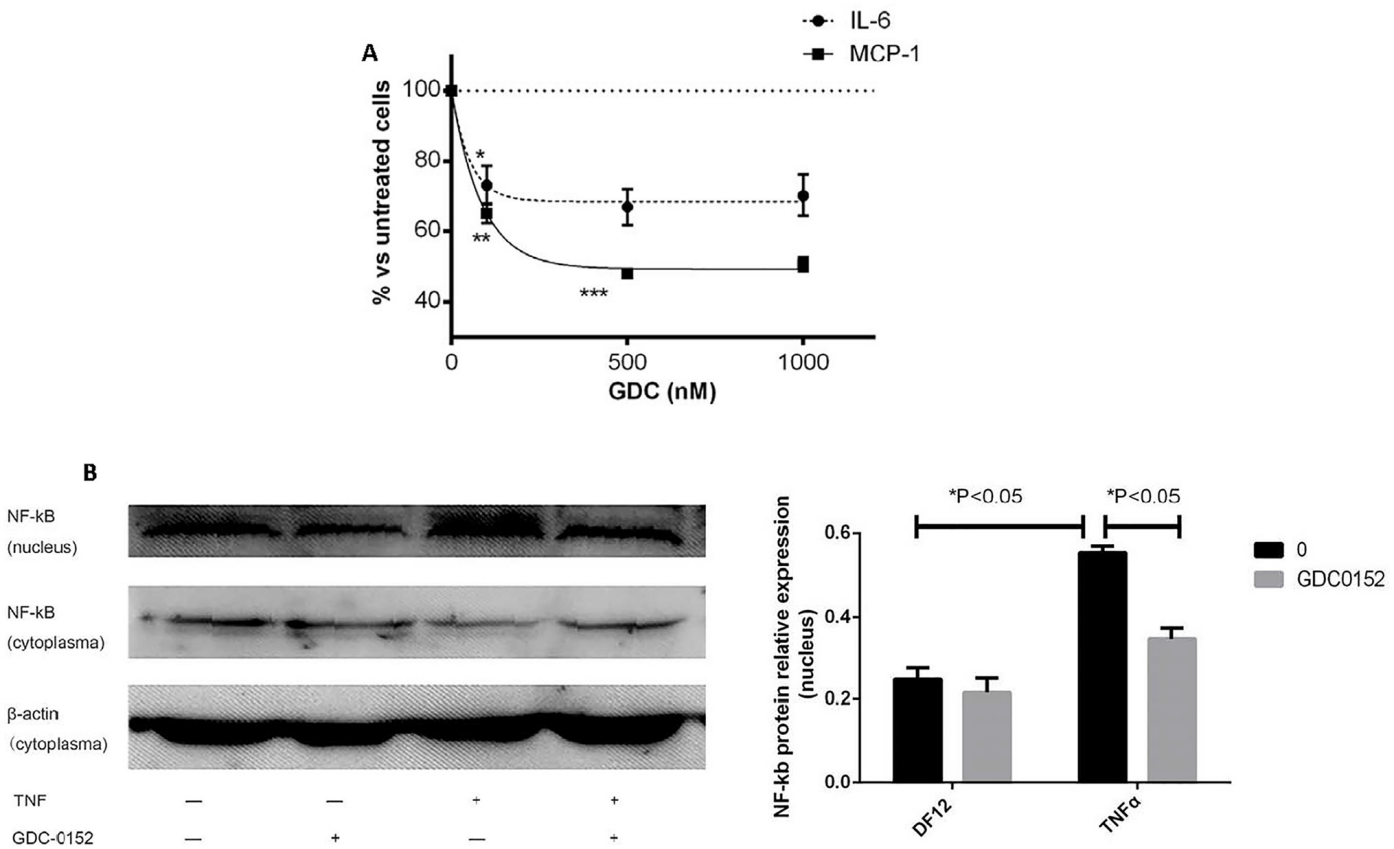
Inhibitor of Apoptosis Proteins (IAPs) were involved in MCP-1/IL-6 production under high dose TNF-α stimulation. A. hUC-MSCs (2x10^4^ in 96-well plates) were pretreated for 2h with the IAP inhibitor GDC-0152 at increasing concentrations (0–1000 nM), then stimulated with TNF-α (20 ng/ml, 1.2 nM). After a further 24h, trypan blue was used to exclude cell toxicity. SN was collected and IL-6 and MCP-1 concentrations were measured by ELISA. B. hUC-MSCs(5×10^5^ in T25 bottle) were pretreated with GDC-0152(1000nM) for 2 h, then stimulated with TNF-α (20 ng/ml, 1.2 nM). 24 hours later, protein from nucleus and cytoplasma were extracted separately and the amount of NF-kB were detected by Western blot. Data are as mean±SEM of triplicate measurements; *p<0.05, **p<0.01, ***p<0.001 when compared to untreated cells. These experiments were repeated 3 times with the same results, using clone 69 and another TNF-α sensitive clone (clone 120003).

MCP-1 and, at a lower level, IL-6 production, appeared associated with cell death induction (Fig 6). However, there was no strict parallelism between the levels of cell death and cytokine production. For example, under the same stimulus, use of the caspase inhibitor zVAD-fmk or the specific inhibitor of RIPK1 kinase activity Necrostatin 1, had no influence on the IL-6/MCP-1 induced production despite its effect on cell death (S3 Fig). The whole series of results showed that cell death was not directly responsible for the IL-6/MCP-1 production, but indirectly linked to a process ending in NF-kB activity. An interaction point was then looked for. In a recent report, Cullen et al suggested that IAPs are required for optimal production of cytokines and chemokines in response to Fas receptor stimulation [14]. We looked at the effect of increasing concentrations (0–1000 nM) of a newly developed small-molecule antagonist of IAPs, GDC-0152 [15] on both MCP-1 and IL-6 production. As shown in Fig 7A, it was observed that 20–30% of the Il-6 production and 40–50% of the MCP-1 production could be inhibited when TNF-α (20 ng/ml, 1.1 nM) was used as the stimulant. A dose-response relationship using increasing TNF-α doses in presence or absence of GDC-0152, showed that the effect of the inhibitor could already be detected at 0.5 ng/ml (0.03 nM) TNF-α. As shown in Fig 7B, TNF-α increase the NF-kB in the nucleus, in contrast, GDC-0152 decreased this process. When TRAIL was the stimulant, the inhibition of IL-6/MCP-1 production was slight, possibly due to the low level of stimulation, but the results were similar to those presented in Fig 7 when associated with TNF-α. In contrast, when IFN-γ was used in the combination, no activity of the inhibitor could be demonstrated. Most importantly, testing the hUC-MSCs responsiveness to a FGF-2 as well as an IL-1ß stimulus clearly showed that, at the GDC-0152 concentrations used, inhibition of IAPs did not increase cell death that could have explained the decreased IL-6/MCP-1 production.

## Discussion

In the present study, we observed that TNF-α, a pleiotropic proinflammatory cytokine, involved in the communication between hUC-MSCs and PBMCs. To determine the precise mechanism, IL-6 and MCP-1 were selected as end-point markers to this interaction. IL-6 is involved in tissue damage repair and regeneration [16, 17] as well as immunosuppressive activities [8, 11, 12]. MCP-1 may play a direct role in anti-inflammation [18, 19] but mainly mobilizes the partners involved in this communication [14, 20]. We have previously reported that IL-1α in part, but essentially IL-1ß (both autocrine and paracrine), as produced by both hUC-MSCs and PBMCs, are the main regulators of IL-6 production by hUC-MSCs [8–10]. Here, we showed that TNF-α production was itself controlled by IL-1ß at the level of PBMCs. This observation agreed with the recent report that IL-1ß treatment increased TNF-α secretion as well as TNF-α signal transduction, by upregulating TNFR1 cell surface expression [21]. However, in our conditions, measuring mRNA, especially TNF-α mRNA, did not give us a clear picture of the mechanisms involved in the production of TNF-α. In contrast to the protein measurements, mRNA measurements by real time quantitative PCR did not provide reliable results, possibly duo to multiple regulation levels of this gene, including transcription mRNA stability or translation. Further studies will be necessary to clarify the protein-mRNA relationships.

Next, we showed that TNF-α played a major role in the regulation of IL-6 and MCP-1 production, through two different signaling pathways. The first one, induced at low levels of TNF-α, was associated with activation of the canonical NF-kB pathway and PI3K, JNK and p38 MAP kinase activities. This stimulating activity was increased when combined with IFN-γ, similar to that observed with IL-1ß, suggesting an amplification of the NF-kB signaling pathway. Another mechanism, induced at high levels of TNF-α, was indirectly linked to cell death and mainly concerned MCP-1 production. This activity was irregular, and depended upon the hUC-MSC clone analyzed. This contrasted with a constant increase of apoptosis when TNF-α was associated with the pro-apoptotic ligand TRAIL or with IFN-γ. The interaction with TRAIL, which is released by epithelial and endothelial cells or neutrophils *in vivo* [22], suggested a complementary function with TNF-α at the level of apoptosis induction. This association could lead to efferocytosis and anti-inflammation. This mechanism should also be considered in the association with IFN-γ although, in this case, cell necrosis, not apoptosis, was involved. This result is at variance with the data of Liu et al. [23] who reported that, in mice, a Fas-mediated but FasL-independent apoptotic process of MSCs was induced by proinflammatory T cells trough IFN-γ enhancement of TNF-α signaling. Finally, similar relationships between chemokine expression, promoting chemotaxis of phagocytes toward apoptotic cells, wound healing and tissue regeneration have been recently reported in other cell types and animal species [14, 24]. Different mechanisms have been proposed to act in these processes. Li et al. reported that caspases 3 and 7 were key players in apoptotic cell death to promote wound healing involving PGE2 [24]. Cullen et al. indicated that RIPK1 protein but not enzyme activity was required in a caspase-independent but IAP-dependent NF-kB stimulation of chemotactic and active factors [14]. Our data agree with this report. Indeed, the effects of the synthetic inhibitor, GDC-0152, pointed to an involvement of IAPs in TNF-α activity. Its use allowed us to show that the canonical NF-kB pathway was completed by another pathway related to cell death, involving IAPs.

**Fig 8:**
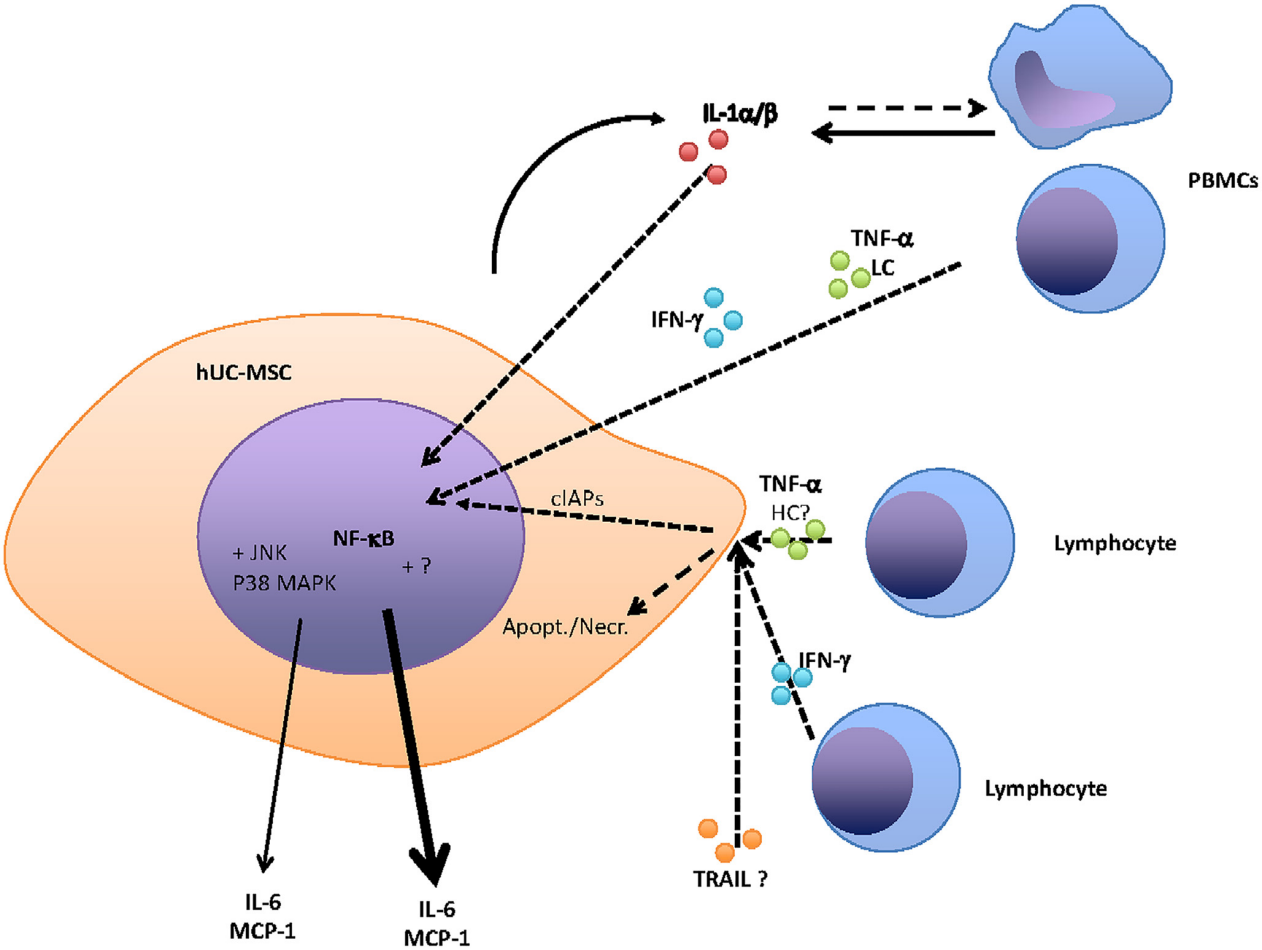
Scheme of interactions between hUC-MSCs and PBMCs. Cytokine receptors are not presented, LC for Low Concentration, HC for High Concentration. Thick full lines are for cytokine production. Dashed line are for cell stimulation.

In summary, (as shown in Fig 8), we defined an important role of TNF-α in the interactions between hUC-MSCs and PBMCs. Il-1ß, which appears to be the master of these interactions, was able to generate TNF-α production from PBMCs. The TNF-α produced was then able to promote IL-6 and MCP-1 production at low concentrations. However, at high concentrations and in association with TRAIL and IFN-γ, TNF-α was also able to induce hUC-MSC death, again associated with IL-6 but mainly with MCP-1 production. The characterization of the local interactions of these crucial factors strongly contributes to the understanding the patho-physiological role of MSCs in the maintenance of tissue homeostasis.

## Supporting Information

S1 Fig Respective effects of cell permeable signaling inhibitors on basal (DF-12 culture medium) and TNF-α -induced IL-6 and MCP-1 production by hUC-MSCs. 2x10^4^ cells in 96-well plates were left untreated or pretreated for 2h with inhibitors of PI3K, c-JNK-1, 2 and 3, MEK1 and MEK2 and p38 MAP kinase, all at a final concentration of 10 μM. Then, cells were stimulated or not with TNF-α (10 ng/ml, 0.6 nM). After a further 48h, trypan blue was used to exclude cell toxicity, supernatants were collected and IL-6 and MCP-1 measured by ELISA. Cell death was not observed. Data, expressed as % of the concentrations measured in absence of inhibitors, are as mean±SEM of triplicate measurements. *<0.05, **<0.01 when compared to untreated cells.
(TIF)

S2 Fig Impact of NF-kB on IL-6 and MCP-1 production. hUC-MSCs (2x10^4^ in 96-well plates) were left untreated or pretreated for 2h with Bay 11–7082 at increasing concentrations (0–500 μM), then stimulated with TNF-α. Cell toxicity was evaluated and SN collected. IL-6 (A) and MCP-1(B) concentrations were measured by ELISA. Wells showing >10% cell death were discarded. They were observed for Bay 11–7082 concentrations higher than 50 μM. Data are mean±SEM of triplicate measurements. This experiment was repeated twice with the same results.
(TIF)

S3 Fig IL-6 and MCP-1 productions are independent of cell death. hUC-MSCs of two different clones (Clones 63 and 69, 2x10^4^ in 96-well plates) were left untreated or pretreated for 2h with zVAD-fmk (V, 20 μM) or necrostatin-1 (C, 50 μM) then stimulated with TNF-α (20 ng/ml, 1.2 nM) associated with TRAIL (500 ng/ml, 28 nM) alone or TNF-α and IFN-γ (50 ng/ml, 3 nM). After a further 24h, cell death was scored by CellTiter-Glo Luminescent Cell Viability Assay. MCP-1 and IL-6 concentrations in SN were measured by ELISA. Data are presented by groups of 3 with the corresponding legend below the x axis, as mean±SEM of six ATP measurements. Representative of 3 different experiments using alternatively clone 63 and 69 with the same results.
(TIF)
