# Supplementary figures and images for: High Concentrations of TNF-α Induce Cell Death during Interactions between Human Umbilical Cord Mesenchymal Stem Cells and Peripheral Blood Mononuclear Cells
Source: PLoS One. 2015 May 29;10(5):e0128647. doi: 10.1371/journal.pone.0128647 (PMC4448993; doi:10.1371/journal.pone.0128647)

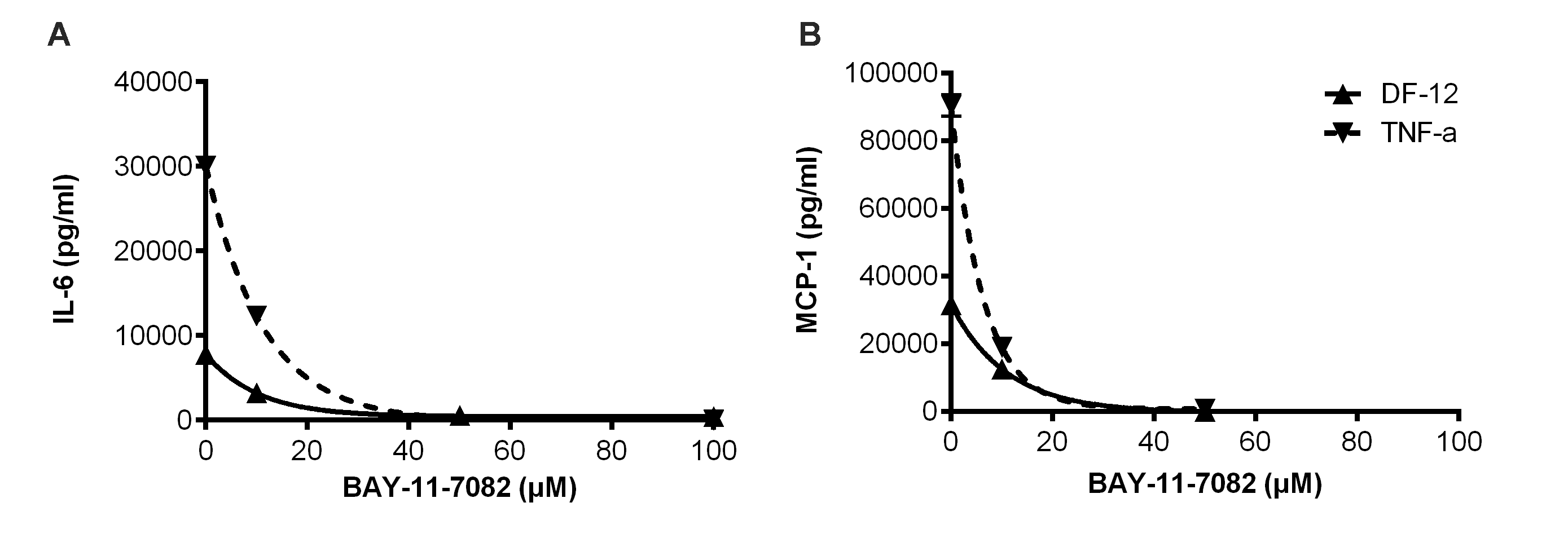

Supplement: S2 Fig — hUC-MSCs (2x10^4^ in 96-well plates) were left untreated or pretreated for 2h with Bay 11–7082 at increasing concentrations (0–500 μM), then stimulated with TNF-α. Cell toxicity was evaluated and SN collected. IL-6 (A) and MCP-1(B) concentrations were measured by ELISA. Wells showing >10% cell death were discarded. They were observed for Bay 11–7082 concentrations higher than 50 μM. Data are mean±SEM of triplicate measurements. This experiment was repeated twice with the same results. (TIF) [file pone.0128647.s002.tif]
